# Pharmacogenetics: An Important Part of Drug Development with A Focus on Its Application

**DOI:** 10.31531/2581-4745.1000111

**Published:** 2018-05-27

**Authors:** JT Oates, D Lopez

**Affiliations:** Department of Pharmaceutical Sciences, Biomanufacturing Research Institute and Technology Enterprise (BRITE), College of Arts and Sciences, North Carolina Central University, USA

**Keywords:** Pharmacogenetics, Pharmacogenomic, Personalized medicine

## Abstract

Since the human genome project in 2003, the view of personalized medicine to improve diagnosis and cure diseases at the molecular level became more real. Sequencing the human genome brought some benefits in medicine such as early detection of diseases with a genetic predisposition, treating patients with rare diseases, the design of gene therapy and the understanding of pharmacogenetics in the metabolism of drugs. This review explains the concepts of pharmacogenetics, polymorphisms, mutations, variations, and alleles, and how this information has helped us better understand the metabolism of drugs. Multiple resources are presented to promote reducing the gap between scientists, physicians, and patients in understanding the use and benefits of pharmacogenetics. Some of the most common clinical examples of genetic variants and how pharmacogenetics was used to determine treatment options for patients having these variants were discussed. Finally, we evaluated some of the challenges of implementing pharmacogenetics in a clinical setting and proposed actions to be taken to make pharmacogenetics a standard diagnostic tool in personalized medicine.

## Introduction

Pharmacogenetics refers to the variability in response to drug therapies in humans, which is a fast-growing area in molecular biology and clinical medicine [1]. The understanding of Mendel’s Laws of inheritance and how gene mutations affect the way we metabolize drugs are the core of this area of science [2]. Pharmacogenetics, a term that became a topic of interest in the 1930s, has now been rediscovered with the introduction of genetic testing [3]. Many genetic and non-genetic factors affect the way we respond to therapies [4]. Genetic factors account for up to 95% in the variations in response to treatment [5]. However, other factors such as cultural, behavioral, and environmental could have an enormous impact on the extent of these variations [5]. An example of genetic factors involved in this process is when we see members of the same family with the same inherited disease (i.e., cystic fibrosis, hypertension) responding differently to the same medical treatment [6]. A genetic alteration known as a single nucleotide polymorphism (SNP) is a change in a nucleotide sequence that alters the pharmacokinetics and pharmacodynamic parameters of drugs [7,8]. Pharmacogenomics tests are directed to identify patients, who are responsive or non-responsive to treatment, develop interactions, produce side effects, and require dosing adjustment [9].

A breakthrough started with the identification of the specific drug-metabolizing enzymes, Cytochrome P450 (CYP450), which are involved in the metabolism and elimination of medicines, detoxification of foreign chemicals, and in the production of cholesterol, steroids, and prostacyclin [10]. Currently, clinicians make therapeutic decisions based on lab tests, biomarkers, and genetic testing [11]. Consequently, there is a need for more reliable genetic testing and recognizable biomarkers to improve patient therapy outcomes. The phenotypes of enzymes are responsible for the inconsistency in dosing therapy (i.e., warfarin dosing), drug activation (i.e., clopidogrel), and drug interactions (i.e., omeprazole and clopidogrel) [12–14].

The concept of personalized medicine has made pharmacogenetics a crucial topic [15]. Personalize medicine refers to the healthcare area where all the information of the patient (uniqueness of a patient) is used to make therapeutic decisions [15]. This information includes genetic, quality of life, and environmental factors [15]. Since 2007, the Food and Drug Administration (FDA), recognizing the need of more clinical information about the use of biomarkers, has released a list of more than 100 drugs with pharmacogenomic information on their labeling and issued a black boxed warning in several of these medications [16]. Table 1 summarizes some of the drugs in that list [16]. Also, pharmaceutical companies acknowledge the importance of personalized medicine to avoid paying for astronomical lawsuits because of fatal side effects [17]. Therefore, they are willing to invest more resources on research in pharmacogenetics and the development of more genetic biomarkers [17].

The 2016 report from the Personalized Medicine Coalition (PMC) reveals that more than 20% of the new molecular entities (NMEs) approved in the U.S. by the FDA are considered personalized medicine [3]. The hypothesis of this project consists in estimating better outcomes in patient’s therapies due to the utilization of pharmacogenetics more extensively in clinical practice. As a result, there is a need for clinicians to become more knowledgeable in the area of pharmacogenetics [3]. For this reason, the purpose of this review is to emphasize the importance of pharmacogenetics in research, discuss the difficulties of implementing genetic testing in medical centers, propose a method to add pharmacogenetic testing during clinical trial phase II and introduce pharmacogenetic as an essential part of the pharmaceutical science programs [3].

## Background

From Mendel in 1865 with the discovery of genes to the Human Genome Project (HGP) in 2003, scientists have been trying to identify the causes of disease at the molecular level [18]. After the HGP ended, new projects have continuously used the genetic sequencing information obtained through that project [18]. Nowadays, we have more details on how our genes influence growth, development, health, and even drug metabolism than ever before [19]. In 2003, the National Human Genome Research Institute (NHGRI) launched a project named the Encyclopedia Of DNA Elements (ENCODE) [20]. The purpose of this project is to identify all functional elements/sequences in the human genome [20]. Since its launching in 2009, next-generation sequencing (NGS) has proven to be a powerful tool in identifying disease-associated variants in many inherited diseases, whereas targeted sequencing is useful in detecting variants in previously known disease-associated genes [21,22]. The Cancer Genome Atlas (TCGA) has increased our understanding of the molecular signatures associated with different types of cancer (23). Also, TCGA has been useful identifying the molecular similarities between different cancer patients and cancer types, as well as documenting the uniqueness of each cancer type [23]. Even, a growing number of organizations and institutions are beginning to develop clinical services and infrastructures supporting pharmacogenetics testing [24,25]. The Pharmacogenomics Research Network (PGRN) consists of three large center-grant projects focusing on enhancing precision medicine through the discovery and translation of genomic variations that influence therapeutic and adverse drug effects in patients [26]. Two of three projects of PGRN are directed to enabling resources for pharmacogenomics and precision medicine [26].

A knowledge PharmGKB base (https://www.pharmgkb.org/) and a PGRN Hub (http://www.pgrn.org/) were established to coordinate the activities of the new PGRN (26).

The most critical limitation in the complete implementation of pharmacogenomics is the accessibility of the genomic data by the research community [27]. For this reason, the creation of database banks such as ClinVar (https://www.ncbi.nlm.nih.gov/clinvar/) and the Novel Materials Discovery (NOMAD) repository (https://nomad-repository.eu/) has been of significant help. ClinVar is a freely accessible, public archive of reports on the relationships among human genetic variations and phenotypes, with supporting evidence [28]. This database provides access to information on how human genetic variation affects the health of patients [28]. ClinVar is hosted by the National Center for Biotechnology Information (NCBI) and funded by the National Institutes of Health (NIH). The Clinical Genome Resource (ClinGen; https://www.clinicalgenome.org/) is also an NIH-funded resource that helps ClinVar to determine the clinical importance of genes and their variants in precision medicine and research [29]. The NOMAD repository contains genetic information of healthy individuals and facilitates the sharing and exchange of results among research groups.

Advances in genomics allow researchers to develop diagnostic tests and drug therapies at faster pace than ever before [15,30]. The ability to sequence DNA and RNA at a fantastic speed and with a low-cost, enable clinicians to make a diagnosis and identify rare disease quickly [21,22,30]. Patients with a rare disease may spend a long time before receiving the correct diagnosis [31,32]. Genetic testing gives patients peace of mind and hope, instead of keeping them in the dark with delayed treatment for their condition [33,34].

The NIH will be starting a new project in the spring of 2018 called “All of Us” (https://allofus.nih.gov/). This project will gather data (from living conditions to genetic information) from at least one million people living in the U.S. to accelerate research and improve health. This project will have a real impact on how precision medicine will be delivered in the future. Precision medicine is the idea of integrating evidence-based health information into the clinical decision-making process and drug therapy treatments [35,36].

Interpreting the data obtained through genetic testing is exceptionally complicated [37]. The scientific community knows that the primary challenge is the interpretation of the results after getting the genetic sequence and identifying a genetic variant [37]. The association of a genetic variation with a specific disease or metabolic pathway is the ultimate goal for genetics [38]. However, the pool of novel variants is enormous, with the potential of having an unknown association with a particular disease [38]. Therefore, genetics need to convert the genomic data into meaningful information for clinicians to make decisions [38]. In a recent survey, physicians responded that they were unsure of how to use pharmacogenetic testing in clinical practice [39,40]. To solve this gap between research and healthcare, researchers and scientists involved in this field need to provide evidence, recommendation, and training to clinicians who lack confidence at the moment of ordering pharmacogenetic testing [36] A physician requires broad information about the patient (integrate medicine) before prescribing a drug based on a specific biomarker [36].

Due to the growing need for pharmacogenomics information and guidance, the FDA has published on their website a list of pharmacogenomic biomarkers in drug labelling [16]. These biomarkers posted on the FDA website include germline or somatic gene variants (polymorphisms, mutations), functional deficiencies with a genetic etiology, gene expression differences, and chromosomal abnormalities [41]. Some of the labels make a recommendation on what the doctor should do based on the biomarkers, which may or may not include pharmacogenetics testing [41].

## Definitions

Pharmacogenetics and pharmacogenomics are terms used interchangeably [42]. However, pharmacogenomics is used more often in the research arena such as genome-wide association studies (GWAS) [43,44]. Pharmacogenomics is the study of the impact of the individual’s genome on his/her response to medication [43,44]. Pharmacogenomics has the potential to change how we approach medicine and drug therapy [44,45]. Pharmacogenetics describes how genes influence the metabolism of drugs [44,46]. The field of pharmacogenetics is aimed to identify patients at a higher genetically-determined risk of adverse effects or an inadequate response to medication [9,44–46].

The purpose of pharmacogenomics is to use genetics to optimize drug therapies, maximize drug efficacy, and minimize adverse drug reactions [43–45]. For this reason, many pharmacogenetic tests have been developed and are valuable in diagnostic and therapeutic [47]. Pharmacogenomics testing is a DNA-based test that detects genetic variations associated with risk of adverse response or drug response [47]. Several important pharmacogenetic tests are currently available through Clinical Laboratory Improvement Amendments (CLIA)-approved laboratories for many years, but their use is limited [24]. The FDA identifies alleles that influence drug effectiveness and toxicity as Pharmacogenetics Biomarkers [16].

Examples of polymorphisms that affect the metabolism of drugs are variants in the gene encoding for the human leukocyte antigen (HLA) system [20]. HLA is the major histocompatibility complex (MHC) in humans, which is comprised of genes located on chromosome 6 [20]. The cell surface glycoproteins encoded by HLA are responsible for presenting antigenic peptides to the T-cell receptor (TCR) on T cells [20]. The HLA complex primary function is to help the immune system differentiate between the body’s proteins and proteins from foreign invaders such as viruses and bacteria [20]. There are multiple variations in the HLA gene that allow each person’s immune system to react to a wide range of foreign invaders [48,49]. Structural mutations in HLA can lead to different clinical responses in patients [48, 49]. Some variations in the HLA gene are responsible for hypersensitivity reactions [50–52]. As a result, before a patient undergoes grafting procedures and blood marrow transplants, the HLA of the donor should match at least six of eight HLA markers to the recipient to decrease the risk of graft-versus-host disease (GVHD) [53].

At least 40 similar alleles have been identified as subtypes of HLA-B27 and are associated with increased risk of developing the inflammatory joint disease known as ankylosing spondylitis and many other disorders involving abnormal immune function [54]. Similarly, to an SNP, variants in the gene that code for HLA influence the way humans metabolize medications [55]. For instance, some variants in the HLA-B*1502 allele are related to carbamazepine-induced Stevens-Johnson syndrome (SJS) and toxic epidermal necrolysis (TEN), which are severe disorders of the skin and mucosal membranes [56]. In SJS, the affected skin dies making the healing process complicated [57]. Many cases of TEN, which is a more severe skin necrosis compared to SJS, have been fatal and in other cases, the healing process could take from months to years [57].

Carbamazepine is indicated for epilepsy, trigeminal neuralgia, and bipolar disorder, and has a higher rate of skin reaction in patients with Chinese ancestry [56,58]. Also, patients who test positive for the HLA-B5701 allele tend to have a severe and life-threatening hypersensitivity reaction to Abacavir (Ziagen) [59]. Currently, a black box warning has been added to the product package insert included with Abacavir indicating that this drug is contraindicated in HLA-B5701-positive patients [59].

Another example is the organic anion transporting polypeptide 1B1 (OATP1B1/SLCO1B1). This transporter, which is mainly expressed at the basolateral membrane of hepatocytes, is involved in the hepatic uptake and clearance of various drug substrates and endogenous compounds including statins [7]. Statins are a class of medication that reduces the production of cholesterol by inhibiting the enzyme 3-hydroxy-3 methylglutaryl coenzyme A (HMG-CoA) reductase [60]. Seven statins are currently on the market [61]. These are atorvastatin, fluvastatin, lovastatin, pitavastatin, pravastatin, rosuvastatin, and simvastatin [61].

Statins are the drug of choice to treat hypercholesterolemia [60,62]. However, they could cause severe side effects like myopathy (muscular weakness and pain) and rhabdomyolysis (irreversible damaged skeletal muscle tissue that breaks down rapidly) [63].

Statins are transported into the liver through OATP1B1 [7,64,65]. Specific variations in OATP1B1 have been associated with reduced internalization of statins into hepatocytes leading to higher levels of the drug in the blood, enhancing the risk of muscle-related side effects [64,65]. One interesting aspect of this transporter is that several of these variations are found in specific ethnic groups explaining why the development of statin-induced muscle issues are more common in some ethnic groups than in others [65].

## Polymorphisms and Pharmacogenetics

Genes are passed on from parents to children, and they determine the genotype and phenotype of a child. Each of us has two copies of each gene, one inherited from each parent. Genes are made up of DNA and instruct cells to make proteins. Human genes vary in size from a few hundred DNA bases to more than 2 million bases. The smallest human genes are those encoding for tRNA’s which 74–93 nucleotides are only long [66]. The largest human genes are the titins, which are involved in muscle ultrastructure and elasticity and code for 3-megadalton proteins [67].

The genetic differences among individuals arise when an alteration in the DNA sequence occurs. This variation of the gene is called allele [41]. Sometimes an allele could have an SNP, which may result, or not, in changes in protein regulation, expression, or activity [41]. SNP are mutations that usually occurs at a frequency of 1% or higher [68]. SNPs can occur every 100–300 base pairs and account for about 90% of all differences in human DNA [68]. Some mutations affect the expression and function of a protein which could be considered as a gain of function or loss of function depending on the specific effect that it has in the metabolic pathway where the protein is involved [68]. These genotypes would affect the individual’s ability to metabolize certain drugs [68]. These individuals could be classified as poor, intermediate, extensive and ultra-rapid metabolizers [69].

SNPs are not the only cause of genetic and phenotypic variation among humans. Through the use of genome-scanning technologies, it has been found the genome also has structural variation (SV) [70]. SVs involve deletions, duplications, insertions, inversions, translocations, and large-scale copy-number variants or copy-number polymorphisms [70]. These SVs seem to make significant contributions to diversity and disease susceptibility in humans [70].

One of the most transforming discoveries for pharmacogenetics and its clinical application was the identification of the CYP450 oxidase that controls the metabolism of debrisoquine and sparteine, CYP2D6 [71,72]. The first polymorphic human drug metabolizing gene, CYP2D6, was cloned and characterized in 1987 [73]. Eventually, polymorphisms in various phase 1 and phase 2 drug metabolizing enzymes and drug transporters were identified and associated with multiple drug response traits [48]. CYP2D6 has been connected to the metabolism of more than 25% of all drugs [71,74]. Since then, more than 80 variants of CYP2D6 have been discovered worldwide, many of which reduce the activity of the enzyme [71,73,75].

These variations are carefully cataloged by the Human CYP Allele Nomenclature Committee, which is now known as the Pharmacogenetic Variation (PharmVar) Consortium (https://www.pharmvar.org/) [74]. Other CYP450 enzymes that have been identified as having a critical role in the metabolism of drugs are CYP2C9 and CPY2C19 [74].

**Figure 1:**
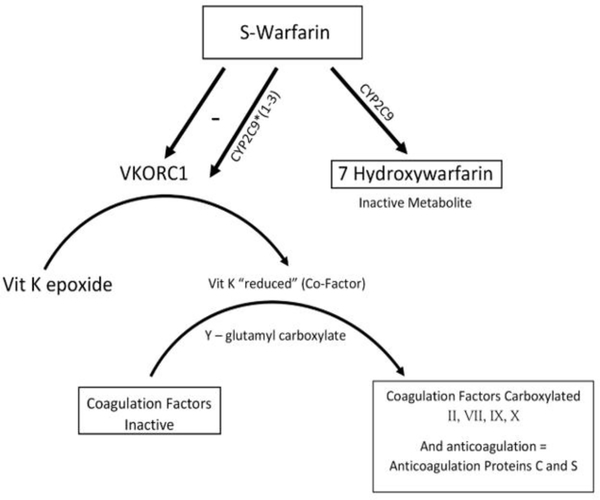
Mode of action of warfarin and the role of SNPs in this process.

Here are some examples of the most well-known and used pharmacogenetic tests applied in clinical practice. First, the anticoagulation effect of warfarin occurs by inhibition in the synthesis of vitamin K-dependent clotting factors (Figure 1) [12,69]. The S-enantiomer of warfarin is mainly hydroxylated by CYP2C9 to its inactive metabolite [12,69,76]. The variant alleles, CYP2C9*2 and CYP2C9*3, result in decreased in vitro CYP2C9 enzymatic metabolism of S-warfarin (poor metabolizer) [12,69,76]. Variants alleles in CYP2C9 are associated with the dose variability and pharmacogenetic of warfarin [76]. This is very important because the S-isoform of warfarin, which is the enantiomer predominantly responsible for the drug’s anticoagulant activity, is metabolized mainly by the CYP2C9 [76]. CYP2C9 converts the drug into the 7-hydroxy and 6-hydroxy inactive metabolites [76]. Patients with one or more variant CYP2C9 alleles have decreased S-warfarin clearance [77,78]. Variability in this enzyme could cause bleeding during warfarin therapy [77,78]. Extensive research has been done referent to the optimal warfarin dosing in patients with genetic polymorphisms [77,78].

The anticoagulant activity of warfarin occurs by inhibiting the enzyme vitamin K epoxide reductase (VKOR) [78]. The role of VKOR is to regenerate vitamin K after oxidation in the gamma-glutamyl carboxylase reaction during stimulation of clotting factors and initiation of the coagulation cascade (Figure 1) [78]. Patient’s therapy is affected by genetic polymorphisms in genes encoding for the enzyme CYP2C9 and warfarin- target enzyme vitamin K epoxide reductase complex 1 (VKORC1) [78–81].

Another example of polymorphic enzymes involved in drug metabolism and their clinically relevant substrates is thiopurine S-methyltransferase (TPMT; also known as thiopurines) [82,83]. Thiopurines, such as azathioprine, mercaptopurine, and thioguanine, are drugs prescribed for diseases like acute lymphoblastic leukemia (ALL), inflammatory bowel disease, and autoimmune disorders [82,84]. These drugs are also be prescribed for organ transplant recipients to prevent organ rejection [82,84].

**Figure 2:**
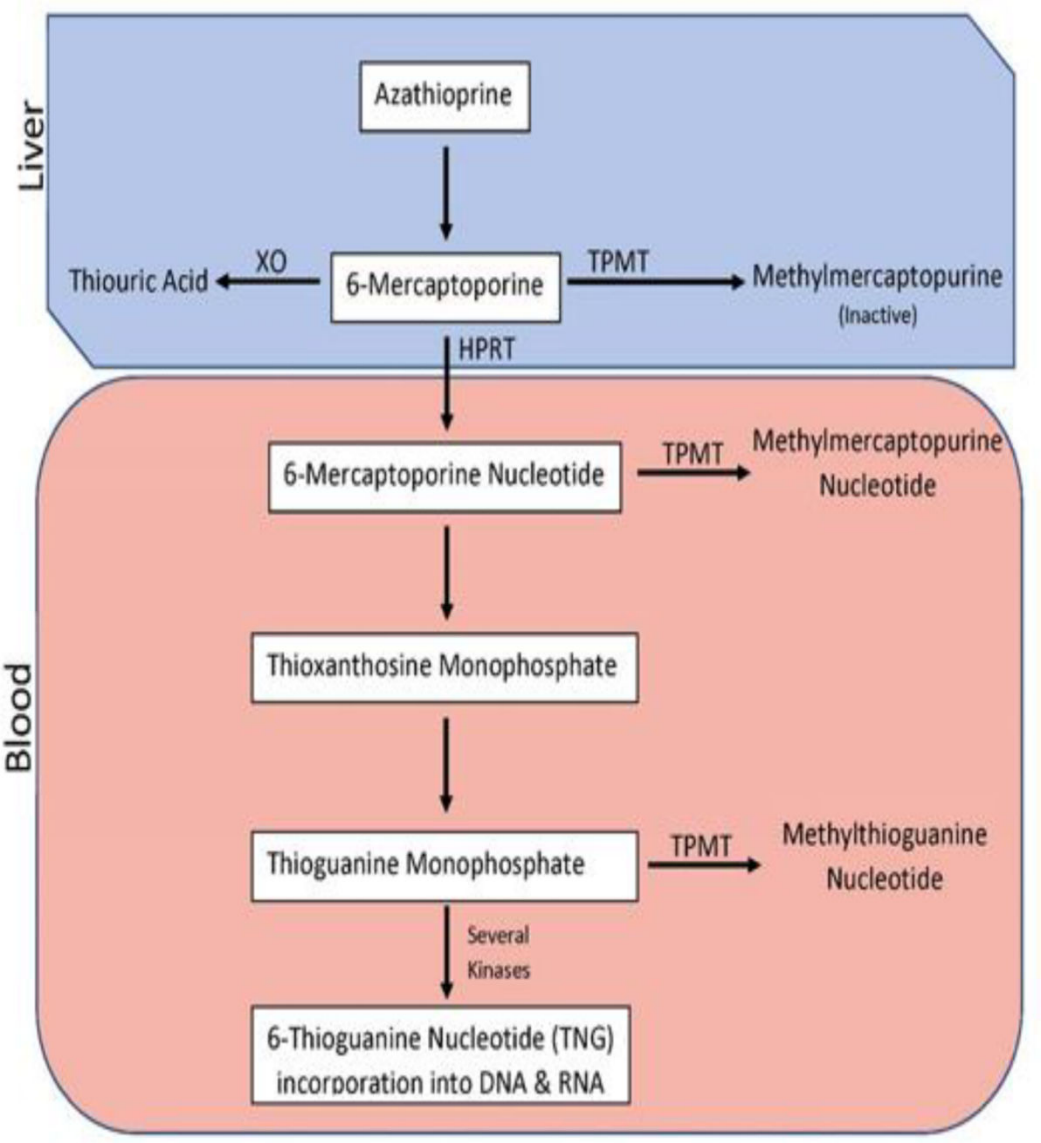
Metabolism of azathioprine.

A TPMT genotype test may be ordered before a person is treated with a thiopurine drug (Figure 2) [83]. Patients with low TPMT activity experiences severe side effects, such as a decrease in white blood cell count [83]. Many of the patients with two wild-type (WT) copies of the TPMT gene have little risk of thiopurine toxicity so that they can be treated with a standard dose of the drug (fast metabolizer) [83]. However, patients who are heterozygous for one WT gene and one genetic variation have decreased TPMT levels [83]. About 30–60% of people who are heterozygous have severe side effects from standard doses of thiopurines so that they will either require reduced doses of the drug or may need an alternative medication [83]. Individuals homozygous for the variant TPMT gene, who have little to no TPMT, are 100% likely to develop severe bone marrow toxicity (myelosuppression) when treated with conventional doses of thiopurines [83]. They must be prescribed an alternative drug [83].

The primary use of pharmacogenetics is in oncology [85]. Pharmacogenetics in oncology is directed to improving the patient’s quality of care [85]. Cancer therapies can cause toxic effects like bone marrow suppression [86]. Pharmacogenetics allows oncologists to personalize treatment based on patients’ individual germline genetic test results [40,85,87]. Also, pharmacogenetics enables the identification of patients who will respond well or not to a particular drug according to their genetic information [40,85,87]. This permits more efficient and prompt drug therapy [40,85,87].

**Figure 3:**
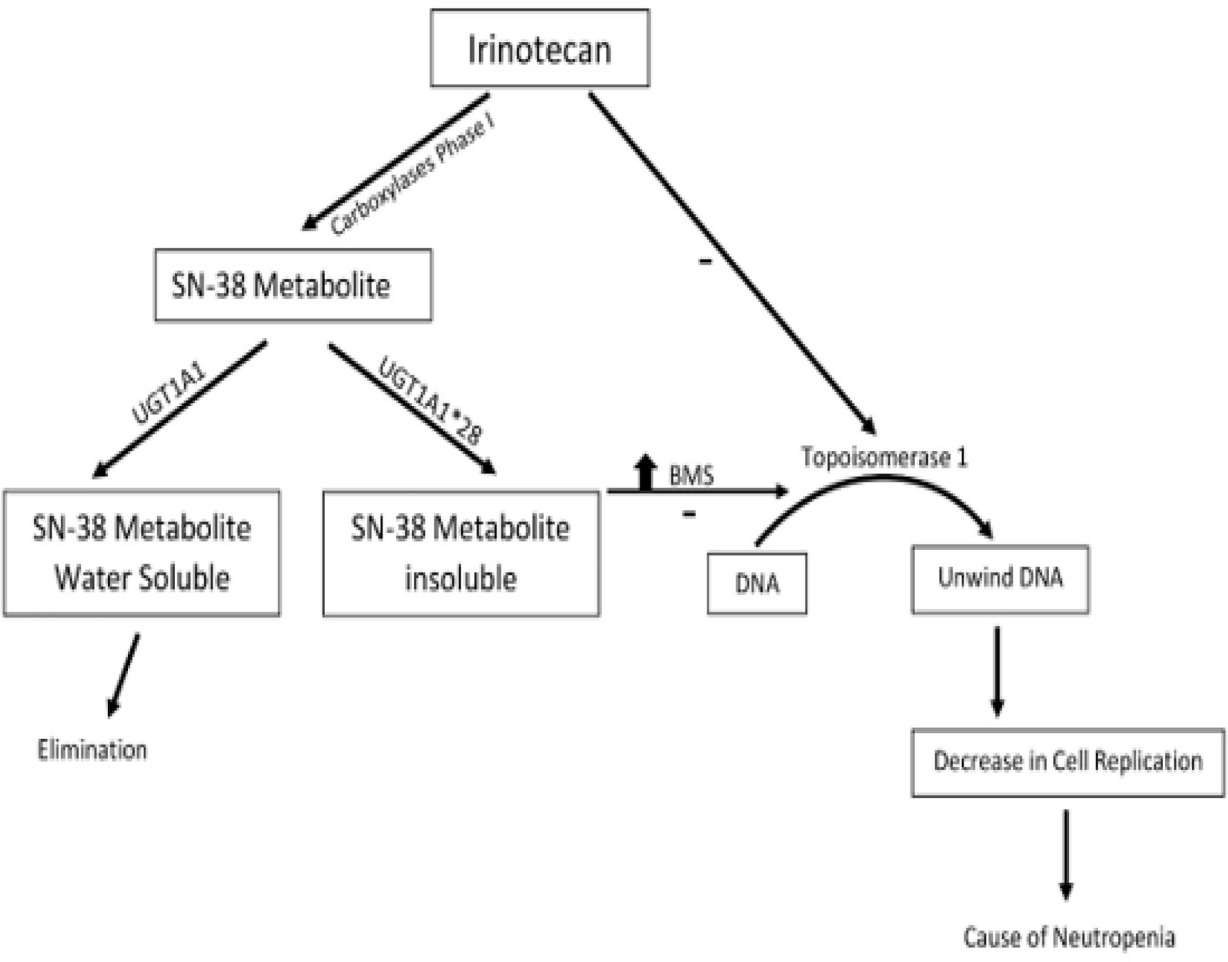
Processing of irinotecan and the effects of different SNPs.

Germline mutations play a significant role in the treatment response to both chemotherapies and targeted anti-cancer agents [88]. There is an association between mutations and the pharmacokinetics of a drug contributing to treatment-related adverse events experienced by patients [88]. One of the applications of pharmacogenetics in oncology is the use of irinotecan, a drug commonly used with 5-Fluorouracil and leucovorin in the treatment of metastatic colon cancer (Figure 3) [89,90]. The active metabolite of irinotecan SN-38 inhibits the topoisomerase I, causing damage to the DNA [89,90]. SN-38 is eliminated from the body through glucuronidation pathway (phase II) [91].

The enzyme uridine diphosphate-glucuronosyltransferase 1A1 (UGT1A1) metabolize SN-38 making it more hydro soluble to be eliminated [91]. Patients who are homozygous for the UGT1A1* 28 allele (UGT1A1 7/7 genotype) are at increased risk for neutropenia (an abnormally low concentration of neutrophils in the blood) [91,92].

Monoclonal antibodies are a class of drug with specific targets and have been a significant advance in patient’s therapy [93]. Trastuzumab (Herceptin) which requires human epidermal growth factor receptor 2 (HER2)-positive protein overexpression to respond well is an example of this type of therapy [94]. HER2 is activated upon forming homodimers or heterodimers with other epidermal growth factor receptor (EGFR) proteins [94].

The dimerization process leads to autophosphorylation of HER2 and phosphorylation of EGFR, which in turn activate several downstream pathways including the Ras/Raf/mitogen-activated protein kinase, the phosphoinositide 3-kinase/Akt, and the phospholipase Cγ (PLCγ)/protein kinase C (PKC) pathways [95]. Also, the dimerization process promotes the mislocalization and rapid degradation of cell cycle inhibitor p27Kip1 protein leading to cell proliferation [95]. Consequently, cancer cells expressing high levels of HER2 are more aggressive and can metastasize easily [95] Although trastuzumab is very effective in treating HER2-positive cancers, many patients usually develop resistance to the treatment mostly due to the production of inactivating antibodies by the patient’s immune system [94].

**Figure 4:**
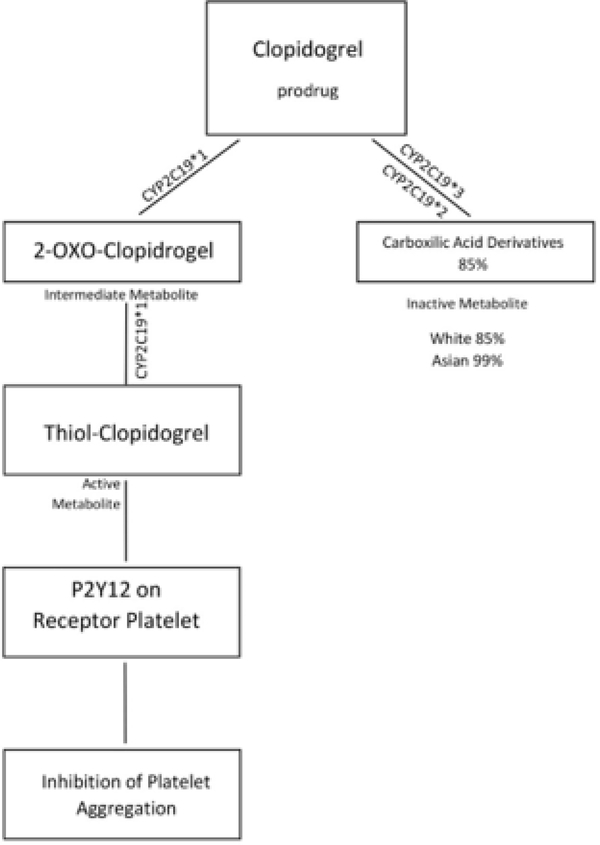
Activation and processing of clopidogrel and the effects of different SNPs.

Plavix (clopidogrel) is an antiplatelet agent that requires bioactivation mediated by CYP2C19 (Figure 4) [96]. Patients who have a CYP2C19 loss-of-function allele have a decreased response to clopidogrel as well as an increased rate of adverse cardiac events and stent thrombosis compared with noncarriers [4,13,96].

Currently, there is convincing evidence to support performing CYP2C19 genotyping in patients with acute coronary syndrome undergoing percutaneous coronary intervention [97]. The dosing recommendations for clopidogrel are based on the interpretation of the CYP2C19 genotype, which is clearly outlined in the Clinical Pharmacogenetics Implementation Consortium Guidelines [97,98].

Polymorphisms in genes that encode for drug transporters and drug targets are shown to alter drug responses [8,55]. Extensive research has been done during the last twenty years to understand the relationship between the genotype and phenotype of these genes [8,55]. Researchers have investigated polymorphisms in several genes including CYPIA1, CYP3A4, CYP3A5, GSTM1, NAT2 and UGT1A1 and lately the OATP1B1/SLCO1B1 [7,46,48,55].

## The Future of Pharmacogenetics

We are in an era when the advances in genetics are changing how we approach diseases and how we treat them. Next-generation sequencing of DNA will continue to develop novel ways to identify genetic factors that affect the way we metabolize drugs and will broaden the boundaries of what we know about the diversity of human genetic by creating genetic databanks accessible to all. With these advances in mind, we can predict some of the changes that will occur in clinical practice. The changes include:

Genomics Education Programme (GEP): these programs include educational materials and training courses available to physicians, pharmacist, and other healthcare practitioners. These programs could be accessed through the site: http://www.westmidsgmc.nhs.uk/tag/genomics-education-programme/.

Pharmacogenetic results will become part of the patient’s medical records to be ready for clinical use when needed. Once genetic test results are in the permanent medical record of a patient, there will be implications for all the agents that are strongly linked to a gene that was found to be mutated. These records should remain relevant for the lifetime of the patient.

Eventually, potentially essential genes that are not often included on commercial genotyping arrays due to homology and structural variation issues (e.g., CYP450 and HLA) [3] will be identified and systematically tested for those genes.

Since HLA-G has immunomodulatory properties [49], the understanding of mechanisms involved in the regulation of this gene may eventually lead to an individualized approach for the future use of HLA for therapeutic purposes.

More complete guidelines will be designed to assist clinicians to understand pharmacogenetic test results and orient the decision-making process to optimize drug’s patient therapy.

## Implementation of Pharmacogenetics

The integration of pharmacogenetic testing into clinical practice has evolved, but not at the same speed as the advances in genetics [2,3,9,27,36,37,42,45]. However, a few notable organizations are leading the way. St. Jude hospital has been the lead hospital in the implementation of pharmacogenomics since they started in the 1990’s [99]. They have their PG4KDS (Pharmacogenomics for Kids) program [99]. Other hospitals have implemented pharmacogenomic testing only on drugs well-known to cause adverse drug events (ADE) [24,100]. In this case, a genetic test is ordered by an authorized physician before initiating drug therapy, and the results of the pharmacogenomic testing are discussed with a group of interdisciplinary clinicians [24,100].

Implementing pharmacogenetics in a clinical setting have many challenges [3,25,35]. A few of the most notable challenges include but are not limited to 1) a weak understanding from clinicians about the clinical utility of the pharmacogenetic test, 2) a lack of confidence in the validity of the genetic tests, 3) difficulties in the interpretation of the test results, 4) the cost of the genetic tests, and 5) the ability to find an alternative treatment [3,25,35].

First, clinicians need a clear understanding and an applicable pathway for the use of genetic testing [35,47,100]. One benefit is the application of genetic principles to guide therapeutic decisions. This approach has a direct implication on patients’ outcomes by maximizing efficacy and decreasing or preventing toxic side effect of drugs. However, this method will require the creation of guidelines with a more extensive explanation about the pharmacogenetic test and the options clinicians have in case a gene variant is confirmed [35,47,100]. A resource to address this need is the Clinical Pharmacogenetics Implementation Consortium (CPIC; https://cpicpgx.org/), which is an international association interested in facilitating the use of pharmacogenetic tests for patient care and personalized medicine [101].

CPIC helps to alleviate the problem of clinical implementation in a healthcare setting by providing peer-reviewed guidelines of how to use pharmacogenetic information [101]. However, the list of drugs addressed currently is limited. Over time, the genomic information could directly connect pharmaceutical companies, physicians, and patients to improve patient’s outcome [101]. This could help improve the identification of drug targets and possibly get medicines to the market quickly by influencing the design of clinical trials [102].

Secondly, there is a lack of confidence regarding the validity of a pharmacogenetic test among clinicians [3,100]. Therefore, they hesitate to proceed with ordering one [3,100]. However, it is essential that clinicians be further educated regarding the difference between analytical validity and clinical validity [3,100]. Analytical validity is the ability of a genetic test to measure the genotype of interest accurately and reliably [103]. On the other hand, clinical validity is the ability of a genetic test to predict the clinical disorder or phenotype associated with the genotype [3,103]. The clinical validity is not always easy to achieve because drugs respond more often to differences in the phenotypes as compared to a genotype [3,103]. In short, the validity of the test has its roots in CLIA, which regulates the overall analytical process hence raising the level of confidence in the test results [103]. Regarding clinical validity, additional research has occurred to correlate these results to specific diseases [103]. However, the application is often limited to particular diseases and associated drugs [103].

Another challenge occurs when practicing clinicians lack applicational understanding in applying pharmacogenetic test results to patient care [105]. As mentioned above, the CPIC guidelines are one resource to assist with this challenge; the information in the FDA website about pharmacogenetics also offers valuable assistance [16,41]. One additional resource is the PGRN [26]. However, there is still an educational gap that currently exists between clinicians and researchers. Although the center of genetic information has been created, we still need specific training for clinicians within the healthcare institutions [106]. Some universities have begun to fill this gap by offering programs, residencies, and fellowships. One of these schools is the University of Florida, which now offers a master’s in clinical pharmaceutical sciences (http://ptr.pharmacy.ufl.edu/education/clinical-pharmaceutical-sciences/).

Also, the Quantitative Biosciences Consortium (QBC; https://qbc.ucsf.edu/) is composed of five Ph.D. graduate programs from the University of California, San Francisco (UCSF). QBC has the purpose of advance education and innovative research at the interface of the biological sciences and the quantitative sciences. Pharmacogenomics is one of the graduate programs included under QBC.

Next, it is probably the challenge that represents the more prevalent attitude against implementing pharmacogenetics in healthcare, which is the cost of genetic testing [107]. The approval and reimbursement for genetic testing by healthcare companies do not facilitate easy access, and the range of fees for such tests can run as much as $2,000 [107]. At this time, only a few tests are covered by insurance with prior approval including, but not limited to, TPMT and familial hypercholesterolemia (FH) tests. However, the approval process could involve multiple requirements and an extended waiting period that could be detrimental to the patient’s health. Many times, patients end up paying themselves for the test to avoid the odyssey of the process [107]. Even after recent revisions of several drug labels by the FDA, now including relevant pharmacogenetic information on them, most of these drugs are not required genetic testing before initiating therapy [108]. Consequently, insurance companies will often refuse to pay for genetic testing indicating a lack of directive regarding whether a genetic test is required or not (108). Therefore, new policies are necessary to expand the use of genetic testing in healthcare settings (108).

The FDA has provided clear guidelines for the submission of pharmacogenomic data [16,109]. These guidelines propose that a pharmacogenomic test result may be considered a valid biomarker as long as [1] it is measured in an analytical test system with well-established controls and [2] there is sufficient scientific evidence that supports the physiologic, pharmacologic, toxicologic, or clinical significance of the test results [16,109]. Examples provided in these guidelines are the connection between CYP2D6 and TPMT to the corresponding drugs that are metabolized by these enzymes [16,109]. These examples are well understood in the scientific community; therefore, appropriate warnings have been added to the individual drug labels [16,109]. There are no clear guidelines on whether a pharmacogenetic test must be done.

For example, the FDA has proposed the value of the approved warfarin pharmacogenetic assay, and the product insert of the drug suggests testing for CYP2C9 and VKORC1 genetic variants to guide warfarin dosing [41,80]. Despite this recommendation by the FDA, the Centers for Medicare and Medicaid determined that it is not reasonable or necessary to perform these tests before prescribing warfarin to patients [110]. Currently, only naïve patients enrolled in a carefully constructed prospective, randomized controlled clinical trial are covered by these two organizations [110]. Therefore, the educational gap also exists within the insurance industry at large. There is a need to identify the clinical utility and possible financial savings that may exist when genetic testing is performed [102,111]. Specifically, the ongoing benefits of pharmacogenetic testing may decrease the cost of hospitalization and healthcare treatment due to ineffective therapies or severe side effects that could have been avoided with genetic testing and tailored medical treatment [102,111]. In fact, most pharmaco-economic studies support the pharmacogenetic testing is a cost-effective or cost-saving strategy [112,113].

Lastly, finding alternative treatments for patients when medications are not effective for a specific disease or condition represents another limitation for implementing pharmacogenetics [87,101]. Taking this into consideration, NIH funded the eMERGE-PGx project [114]. This project utilizes electronic medical records and a research infrastructure from participating healthcare systems to commence pre-emptive pharmacogenetic testing to evaluate clinical outcomes and other areas of clinical implementation.

## Pharmacogenomics and Drug Discovery

Notable advances in genome sequencing technology have allowed pharmaceutical companies to develop sophisticated molecular models, with adequate computational and informatics support [115]. The data show that researchers are twice as likely to find a drug target using genomic information and computational assistance than through old methods [107, 116]. Genetics could help improve the identification of drug targets and possibly get medicines to the market more quickly by influencing the design of clinical trials [117]. Moreover, genetics could help Pharmaceutical companies in predicting drug response based upon personalized clinical variants during clinical trials [117]. Also, researchers are interested in discovering and validating tests for eventual clinical use. The first step is to associate the gene with the disease, then scientists from different areas of the investigation come together to obtain a molecule that they can modify to treat the condition or disease [118]. The advances in genetics impact the development of new drugs because we gain a more accurate understanding of diseases and the pharmacokinetic and pharmacodynamics of the new drug [117].

Targets that succeed during the target validation process are also more likely to be genetically validated [119,120]. However, as of today, only 10 to 15 % of the targets have genetic data on them [119,120]. This is important because genetically validated drugs result in a reduction in the cost [119,120]. A candidate drug is more likely to correct the biochemical defect increasing the chance of been approve for commercial marketing [119,120]. By having an understanding of human genetics, less expensive clinical trials are designed by reducing the time and cost of data collection [121]. Also, the therapeutic risk and benefit ratio is optimized by reducing side effects in patients during clinical trials [121]. Human genetics provides crucial information and clinical evidence for drug discovery and research, to move confidently to the next stage of the process [121]. This would give scientists the ability to select the most responsive patients for the clinical study, with the best chance for success and provide reliability to the drug development process [121]. Researchers are walking into a new era of medicine in which research, technology, and new policies making patients, researchers, and providers work together toward the development of precision medicine [3,36,46].

## Pharmacogenetics in Clinical Trials

**Figure 5:**
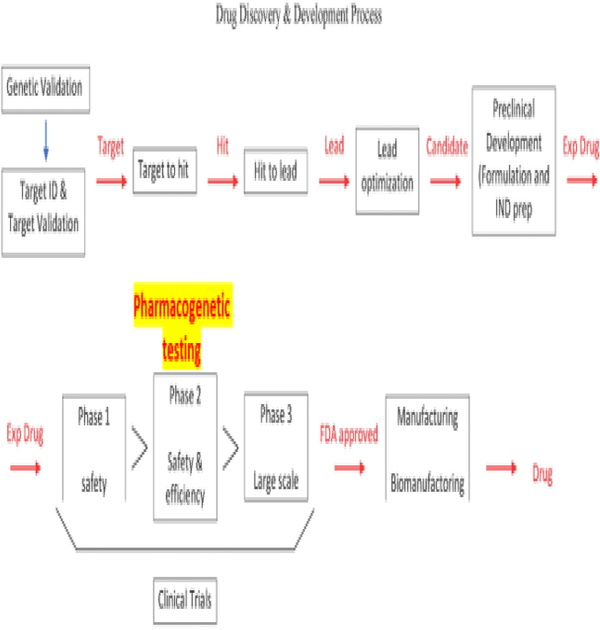
Proposal to include pharmacogenetic testing as part of Phase 2 clinical trials.

Considering the guidelines proposed by the FDA for the submission of pharmacogenomic data (described above), this organization has also created the Biomarker Qualification Program that facilitates the interaction between the Center for Drug Evaluation and Research (CDER) with stakeholders to support the drug development process [122]. Biomarkers are used in research, drug development, and now, in pharmacogenetic testing [11,122,123]. Identifying genes that encode variants as biomarkers are the goal of genetics [11,122,123]. These variants could 1) affect how the drug is activated, transported, or metabolized, 2) act in alternative pathways to those affected by the drug, 3) lead to the development of adverse effects or toxicity once the drug is used, or 4) be directly involved in disease acquisition and progression. We recommend adding pharmacogenetic testing as part of phase 2 clinical trials (Figure 5) so that we can better interpret the results for safety and efficacy of the drug. As more studies are done using pharmacogenetics, the probabilities of developing more efficient, safer, personalized drugs are increased. Furthermore, this could lead to promoting the development of guidelines to implement pharmacogenetics in the clinical setting.

## Conclusion

**Figure 6:**
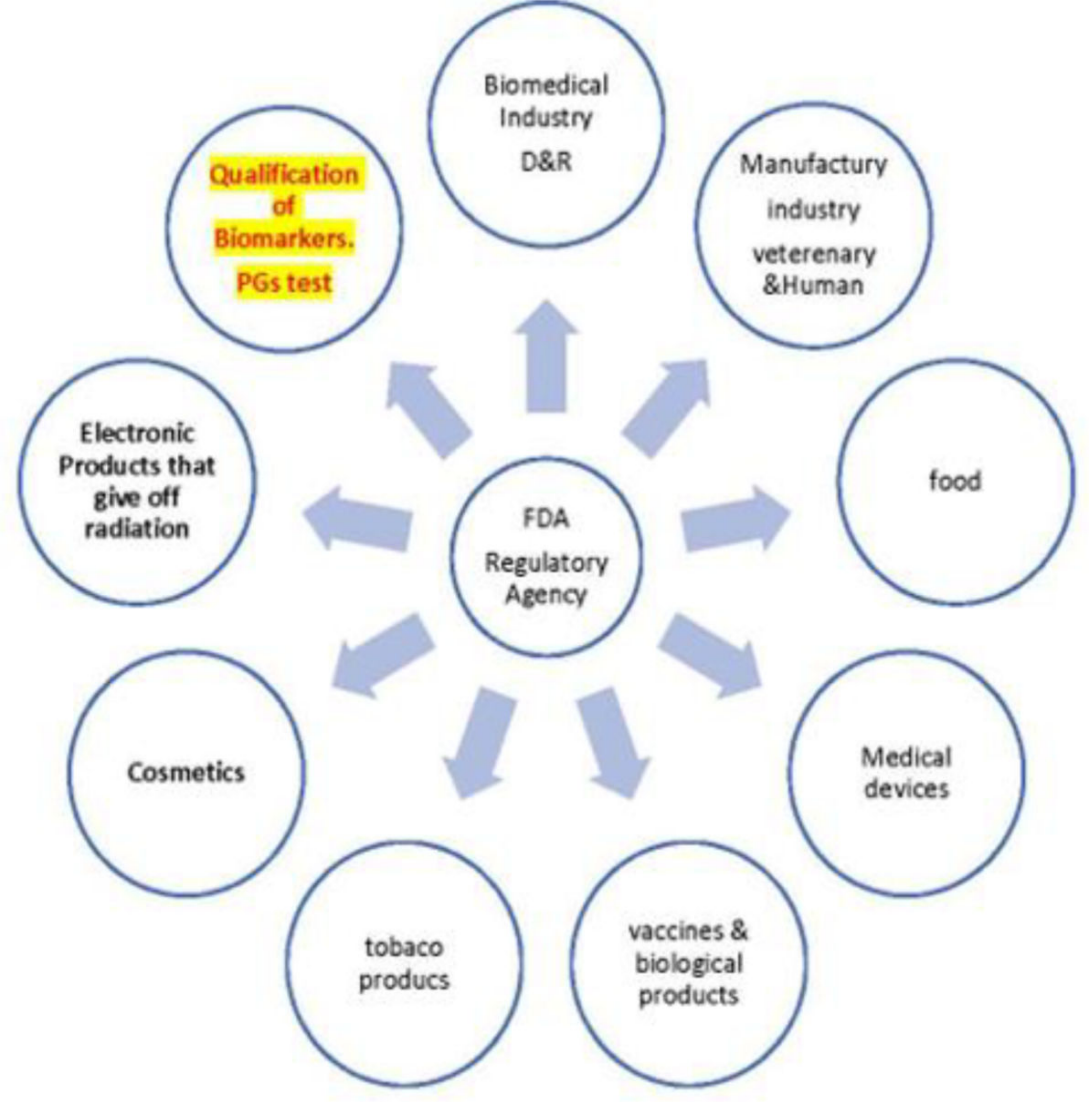
Areas regulated by the FDA and under which section pharmacogenetics (PGs) testing should be regulated.

Using pharmacogenomics to drive the selection of better drug targets is the primary aim of Open Targets [124]. This site has been designed to assist in identification and prioritization of biological targets to be used in drug discovery [124]. The link between a target and a specific disease is backed using integrated genome-wide data collected from different sources [124]. This is critical if we want to implement pharmacogenetics as an essential part of diagnosis and therapeutic decisions and improve the drug discovery process. The FDA has incorporated pharmacogenetics information into drug labels and now offers a list of drugs linked to genetic biomarkers emphasizing the importance of pharmacogenetics for personalized medicine [16,41]. However, this government agency should work in the development of clear guidelines that will lead to the widespread implementation of pharmacogenetics not only during the drug development process but also in the clinical setting to make diagnostic and treatment decisions. Patients should not suffer or even die because insurance companies would not pay for currently available pharmacogenetic tests that would help them qualify for better drugs or for a drug dose that will not cause harm to them. Pharmacogenetic testing should be regulated under the FDA section dedicated to the qualification of biomarkers (Figure 6). Also, there should be clear directions, and not suggestions, for pharmacogenetic testing. Much work still needs to be done, but we are going in the right direction.

## Additional Resources

- Here is a list of resources that could be accessed to learn more about the importance of pharmacogenomics in personalized medicine:
- PharmGKB (https://www.pharmgkb.org/): a site for find information about drugs, pathways, recommended dosings, and drug labels. This site also provides information on pharmacogenetics and how to implement it.
- Ensembl (http://www.ensembl.org/index.html): a genome browser that supports research in comparative genomics, evolution, sequence variation and transcriptional regulation. Three different genomes are included in this browser.
- KEGG: Kyoto Encyclopedia of Genes and Genomes (http://www.genome.jp/kegg/): a database including genome sequences that could be used to understand the functions of biological systems.
- Bioconductor (https://www.bioconductor.org/): a software tool to analyze genomic data.
- National Institute of General Medical Sciences (https://www.nigms.nih.gov/education/Pages/factsheet-pharmacogenomics.aspx): a learning site about pharmacogenomics and how to use it in the drug industry.
- PubMed (https://www.ncbi.nlm.nih.gov/pubmed/): a site to find articles about different scientific subjects including pharmacogenetics.
- The American Society of Human Genetics (http://www.ashg.org/): a primary professional membership organization for human genetics specialists worldwide that allows the exchange of information among experts in pharmacogenetics.
- Pharmacogenomics Research Network (http://www.pgrn.org/): an organization that promotes research in precision medicine that involves the study of pharmacogenetics.

## Figures and Tables

**Table 1:** Summary of some of the drugs in the FDA list.

Drug	Testing and/or Recommendations	Effect and Considerations
Abacavir	HLA-B*5701	If test positive, do not use abacavir
Clopidogrel	CYP2C19 genotype	Consider alternative treatment in patients identified as CYP2C19 poor metabolizers (have 2C19*2 or *3 alleles).
Carbamazepine	HLA-B*1502 in Asian patients	If test positive, do not use carbamazepine unless benefit clearly outweighs the risk.
Trastuzumab Lapatinib Pertuzumab	HER2 protein overexpression	Must be positive (2+ or 3+) to use the drug
Cetuximab	KRAS	If positive for a KRAS mutation on codon 12 or 13, do not use drugs.
Erlotinib	EGFR	If EGFR protein positive, they can use these drugs.
Imatinib	Kit (CD117)	If positive, they can use the drug
Imatinib Dasatinib Nilotinib PONATinib Bosutinib	BCR-ABL	Must be BCR-ABL positive to use the drug
Imatinib	Platelet-derived growth factor receptor (PDGFR)	If PDGFR gene rearrangement is positive, they can use the drug
Maraviroc	HIV tropism with Trofile test	If CCR5-positive, they can use the drug
Rituximab	B-cell CD20 Expression	If positive, they can use the drug
Azathioprine Mercaptopurine	Thiopurine methyltransferase (TPMT)	If positive for loss of function TPMT allele, find an alternative treatment or start at a very low dose
Ivacaftor	CFTR G551D, G1244E, G1349D, G178R, G551S, S1251N, S1255P,S549N, S549R, R117H mutation carriers	If positive, can use the drug
Allopurinol	HLA-B*5801	Consider testing before starting therapy in high-risk individuals (Korean patients with significant renal impairment or those of Han Chinese or Thai ancestry).
Codeine	CYP2D6	Per CPIC recommendations: In CYP2D6 ultra-rapid metabolizers, avoid codeine due to the potential for toxicity. In CYP2D6 poor metabolizers, avoid codeine due to lack of efficacy.
Warfarin	CYP 2C9 and VKORC1	If not testing, use caution by selecting a low starting dose, increasing slowly, and monitoring INR frequently. If the test indicates variations, a safer, lower starting dose can be selected.
Capecitabine	Dihydropyrimidine dehydrogenase (DPD) deficiency	If positive, do not use the drug
Phenytoin Fosphenytoin	HLA-B*1502 for Asian patients	If positive, do not use unless benefit clearly outweighs the risk.
Irinotecan	UGT1A1*28	If positive, consider reducing the dose
Tamoxifen	Estrogen receptor 1	If positive, they can use the drug
